# 2-Chloro-*N*′-(2,4-dichloro­benzyl­idene)benzohydrazide

**DOI:** 10.1107/S1600536810007531

**Published:** 2010-03-06

**Authors:** Cong-Shan Zhou, Tao Yang

**Affiliations:** a College of Chemistry and Chemical Engineering, Hunan Institute of Science and Technology, Yueyang, Hunan 414006, People’s Republic of China

## Abstract

The title Schiff base compound, C_14_H_9_Cl_3_N_2_O, exists in a *trans* configuration with respect to the C=N bond and the dihedral angle between the two benzene rings is 13.5 (2)°. In the crystal, inter­molecular N—H⋯O hydrogen bonds link adjacent mol­ecules into extended *C*(4) chains propagating along the *c*-axis direction.

## Related literature

For a related structure and background material, see the previous paper: Zhou & Yang (2010 ▶).
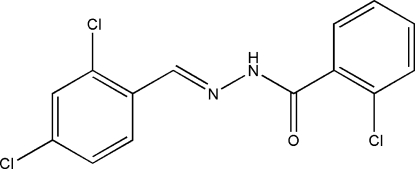


## Experimental

### 

#### Crystal data

C_14_H_9_Cl_3_N_2_O
*M* *_r_* = 327.58
Monoclinic, 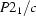

*a* = 7.4737 (11) Å
*b* = 25.877 (4) Å
*c* = 8.1833 (12) Å
β = 116.013 (2)°
*V* = 1422.3 (4) Å^3^
*Z* = 4
Mo *K*α radiation
μ = 0.64 mm^−1^
*T* = 298 K
0.23 × 0.20 × 0.20 mm

#### Data collection

Bruker SMART 1000 CCD diffractometer
Absorption correction: multi-scan (*SADABS*; Sheldrick, 1996 ▶) *T*_min_ = 0.867, *T*_max_ = 0.883
7752 measured reflections
2828 independent reflections
2066 reflections with *I* > 2σ(*I*)
*R*_int_ = 0.029

#### Refinement

*R*[*F* ^2^ > 2σ(*F* ^2^)] = 0.044
*wR*(*F* ^2^) = 0.121
*S* = 1.02
2828 reflections
184 parameters
1 restraint
H atoms treated by a mixture of independent and constrained refinement
Δρ_max_ = 0.59 e Å^−3^
Δρ_min_ = −0.36 e Å^−3^

### 

Data collection: *SMART* (Bruker, 2007 ▶); cell refinement: *SAINT* (Bruker, 2007 ▶); data reduction: *SAINT*; program(s) used to solve structure: *SHELXTL* (Sheldrick, 2008 ▶); program(s) used to refine structure: *SHELXTL*; molecular graphics: *SHELXTL*; software used to prepare material for publication: *SHELXTL*.

## Supplementary Material

Crystal structure: contains datablocks global, I. DOI: 10.1107/S1600536810007531/hb5344sup1.cif

Structure factors: contains datablocks I. DOI: 10.1107/S1600536810007531/hb5344Isup2.hkl

Additional supplementary materials:  crystallographic information; 3D view; checkCIF report

## Figures and Tables

**Table 1:** Hydrogen-bond geometry (Å, °)

*D*—H⋯*A*	*D*—H	H⋯*A*	*D*⋯*A*	*D*—H⋯*A*
N2—H2⋯O1^i^	0.89 (1)	2.00 (1)	2.864 (3)	164 (3)

Symmetry code: (i) 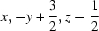.

**Fig. 1.**
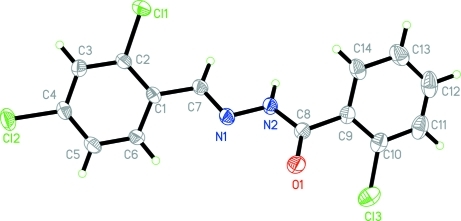
The molecular structure of (I), with ellipsoids drawn at the 30% probability level.

**Fig. 2.**
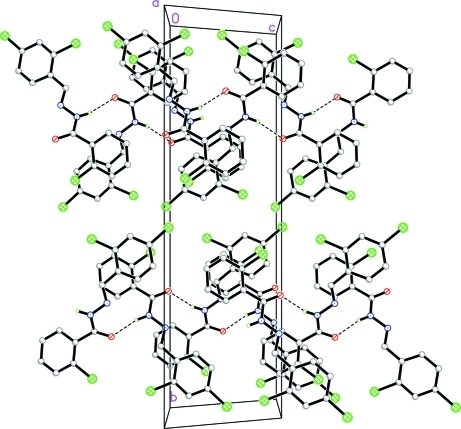
The packing of (I), viewed along the a axis. Hydrogen bonds are drawn as dashed lines.

## supplementary crystallographic information

### Comment

As part of our ongoing studies of Schiff bases (Zhou & Yang, 2010), the crystal structure of the title Schiff base, (I), derived from the condensing of 2,4-dichlorobenzaldehyde with 2-chlorobenzohydrazide in methanol is reported.

The molecule exists in a *trans* configuration with respect to the acyclic C═N bond. The molecule of the compound is distorted, with the dihedral angle between the two benzene rings of 13.5 (2)°.

In the crystal structure, intermolecular N—H···O hydrogen bonds link adjacent molecules into extended chains along the *c* axis (Table 1 and Fig. 2).

### Experimental

2,4-Dichlorobenzaldehyde (1.0 mmol, 175 mg) and 2-chlorobenzohydrazide (1.0 mmol, 170 mg) were dissolved in a methanol solution (30 ml). The mixture was stirred for 30 min at room temperature. The resulting solution was left in air for a few days, yielding colourless blocks of (I).

### Refinement

H2 attached to N2 was located in a difference map and refined with N—H distance restrained to 0.90 (1)Å. The remaining H atoms were positioned geometrically, with C—H distances of 0.93 Å, and refined using a riding model, with *U*_iso_(H) = 1.2*U*_eq_(C).

### Crystal data

**Table:**

C_14_H_9_Cl_3_N_2_O	*F*(000) = 664
*M**_r_* = 327.58	*D*_x_ = 1.530 Mg m^−^^3^
Monoclinic, *P*2_1_/*c*	Mo *K*α radiation, λ = 0.71073 Å
Hall symbol: -P 2ybc	Cell parameters from 2111 reflections
*a* = 7.4737 (11) Å	θ = 3.0–24.9°
*b* = 25.877 (4) Å	µ = 0.64 mm^−^^1^
*c* = 8.1833 (12) Å	*T* = 298 K
β = 116.013 (2)°	Block, colourless
*V* = 1422.3 (4) Å^3^	0.23 × 0.20 × 0.20 mm
*Z* = 4	

### Data collection

**Table:**

Bruker SMART 1000 CCD diffractometer	2828 independent reflections
Radiation source: fine-focus sealed tube	2066 reflections with *I* > 2σ(*I*)
graphite	*R*_int_ = 0.029
ω scans	θ_max_ = 26.2°, θ_min_ = 2.9°
Absorption correction: multi-scan (*SADABS*; Sheldrick, 1996)	*h* = −9→7
*T*_min_ = 0.867, *T*_max_ = 0.883	*k* = −28→32
7752 measured reflections	*l* = −10→9

### Refinement

**Table:**

Refinement on *F*^2^	Primary atom site location: structure-invariant direct methods
Least-squares matrix: full	Secondary atom site location: difference Fourier map
*R*[*F*^2^ > 2σ(*F*^2^)] = 0.044	Hydrogen site location: inferred from neighbouring sites
*wR*(*F*^2^) = 0.121	H atoms treated by a mixture of independent and constrained refinement
*S* = 1.02	*w* = 1/[σ^2^(*F*_o_^2^) + (0.0516*P*)^2^ + 0.7512*P*] where *P* = (*F*_o_^2^ + 2*F*_c_^2^)/3
2828 reflections	(Δ/σ)_max_ = 0.001
184 parameters	Δρ_max_ = 0.59 e Å^−^^3^
1 restraint	Δρ_min_ = −0.36 e Å^−^^3^

### Special details

**Table:**

Geometry. All esds (except the esd in the dihedral angle between two l.s. planes) are estimated using the full covariance matrix. The cell esds are taken into account individually in the estimation of esds in distances, angles and torsion angles; correlations between esds in cell parameters are only used when they are defined by crystal symmetry. An approximate (isotropic) treatment of cell esds is used for estimating esds involving l.s. planes.
Refinement. Refinement of F^2^ against ALL reflections. The weighted R-factor wR and goodness of fit S are based on F^2^, conventional R-factors R are based on F, with F set to zero for negative F^2^. The threshold expression of F^2^ > 2sigma(F^2^) is used only for calculating R-factors(gt) etc. and is not relevant to the choice of reflections for refinement. R-factors based on F^2^ are statistically about twice as large as those based on F, and R- factors based on ALL data will be even larger.

### Fractional atomic coordinates and isotropic or equivalent isotropic displacement parameters (Å^2^)

**Table:**

	*x*	*y*	*z*	*U*_iso_*/*U*_eq_	
Cl1	0.19289 (14)	0.55576 (3)	0.33995 (10)	0.0770 (3)	
Cl2	0.73603 (12)	0.52632 (3)	1.02156 (11)	0.0691 (3)	
Cl3	0.07212 (14)	0.90788 (3)	0.33033 (13)	0.0715 (3)	
H2	0.076 (5)	0.7358 (12)	0.209 (2)	0.080*	
N1	0.1947 (3)	0.71586 (8)	0.4668 (3)	0.0436 (5)	
N2	0.0880 (3)	0.74638 (8)	0.3170 (3)	0.0435 (5)	
O1	0.0219 (3)	0.80567 (7)	0.4865 (2)	0.0554 (5)	
C1	0.3509 (4)	0.63513 (10)	0.5759 (3)	0.0395 (6)	
C2	0.3470 (4)	0.58197 (10)	0.5493 (3)	0.0438 (6)	
C3	0.4627 (4)	0.54838 (10)	0.6852 (4)	0.0477 (7)	
H3	0.4554	0.5129	0.6648	0.057*	
C4	0.5895 (4)	0.56846 (11)	0.8522 (3)	0.0450 (6)	
C5	0.6012 (4)	0.62076 (11)	0.8836 (4)	0.0482 (7)	
H5	0.6895	0.6339	0.9961	0.058*	
C6	0.4815 (4)	0.65335 (10)	0.7478 (3)	0.0444 (6)	
H6	0.4874	0.6887	0.7707	0.053*	
C7	0.2298 (4)	0.67024 (10)	0.4305 (3)	0.0421 (6)	
H7	0.1777	0.6593	0.3102	0.051*	
C8	0.0076 (4)	0.79055 (9)	0.3394 (3)	0.0419 (6)	
C9	−0.1100 (4)	0.81908 (10)	0.1658 (3)	0.0421 (6)	
C10	−0.0930 (4)	0.87193 (11)	0.1520 (4)	0.0510 (7)	
C11	−0.2088 (6)	0.89765 (13)	−0.0095 (5)	0.0675 (9)	
H11	−0.1944	0.9331	−0.0191	0.081*	
C12	−0.3438 (6)	0.87046 (16)	−0.1536 (5)	0.0769 (11)	
H12	−0.4230	0.8879	−0.2606	0.092*	
C13	−0.3653 (5)	0.81802 (15)	−0.1446 (4)	0.0701 (10)	
H13	−0.4571	0.8003	−0.2454	0.084*	
C14	−0.2510 (4)	0.79142 (13)	0.0140 (3)	0.0557 (8)	
H14	−0.2663	0.7559	0.0211	0.067*	

### Atomic displacement parameters (Å^2^)

**Table:**

	*U*^11^	*U*^22^	*U*^33^	*U*^12^	*U*^13^	*U*^23^
Cl1	0.0824 (6)	0.0591 (5)	0.0504 (5)	0.0065 (4)	−0.0068 (4)	−0.0140 (4)
Cl2	0.0573 (5)	0.0710 (5)	0.0564 (5)	0.0074 (4)	0.0042 (4)	0.0240 (4)
Cl3	0.0819 (6)	0.0509 (4)	0.0903 (6)	−0.0115 (4)	0.0457 (5)	−0.0095 (4)
N1	0.0532 (13)	0.0462 (13)	0.0320 (11)	0.0056 (10)	0.0192 (10)	0.0040 (9)
N2	0.0597 (14)	0.0440 (12)	0.0296 (11)	0.0123 (10)	0.0221 (11)	0.0046 (9)
O1	0.0890 (15)	0.0477 (11)	0.0363 (10)	0.0098 (10)	0.0336 (10)	0.0001 (8)
C1	0.0392 (14)	0.0470 (14)	0.0319 (13)	0.0031 (11)	0.0150 (11)	0.0036 (11)
C2	0.0406 (15)	0.0472 (15)	0.0359 (13)	0.0019 (12)	0.0098 (12)	−0.0017 (11)
C3	0.0454 (16)	0.0423 (14)	0.0488 (16)	0.0026 (12)	0.0146 (13)	0.0042 (12)
C4	0.0354 (14)	0.0539 (16)	0.0402 (14)	0.0002 (12)	0.0116 (12)	0.0112 (12)
C5	0.0448 (16)	0.0601 (18)	0.0333 (13)	−0.0106 (13)	0.0111 (12)	0.0018 (12)
C6	0.0513 (16)	0.0429 (14)	0.0354 (13)	−0.0045 (12)	0.0156 (12)	−0.0007 (11)
C7	0.0495 (16)	0.0469 (15)	0.0302 (13)	0.0044 (12)	0.0177 (12)	0.0002 (11)
C8	0.0527 (16)	0.0413 (14)	0.0368 (13)	0.0014 (12)	0.0244 (12)	0.0023 (11)
C9	0.0534 (16)	0.0450 (14)	0.0378 (14)	0.0112 (12)	0.0291 (13)	0.0059 (11)
C10	0.0623 (18)	0.0494 (16)	0.0565 (17)	0.0091 (13)	0.0401 (15)	0.0056 (13)
C11	0.089 (3)	0.0591 (19)	0.072 (2)	0.0241 (18)	0.052 (2)	0.0227 (17)
C12	0.093 (3)	0.093 (3)	0.058 (2)	0.045 (2)	0.045 (2)	0.029 (2)
C13	0.069 (2)	0.096 (3)	0.0420 (17)	0.0223 (19)	0.0210 (16)	−0.0035 (17)
C14	0.0590 (18)	0.076 (2)	0.0337 (14)	0.0234 (15)	0.0219 (14)	0.0061 (13)

### Geometric parameters (Å, °)

**Table:**

Cl1—C2	1.729 (3)	C5—C6	1.369 (4)
Cl2—C4	1.726 (3)	C5—H5	0.9300
Cl3—C10	1.714 (3)	C6—H6	0.9300
N1—C7	1.272 (3)	C7—H7	0.9300
N1—N2	1.380 (3)	C8—C9	1.497 (3)
N2—C8	1.341 (3)	C9—C10	1.383 (4)
N2—H2	0.893 (10)	C9—C14	1.420 (4)
O1—C8	1.225 (3)	C10—C11	1.391 (4)
C1—C2	1.391 (4)	C11—C12	1.364 (5)
C1—C6	1.396 (3)	C11—H11	0.9300
C1—C7	1.454 (3)	C12—C13	1.372 (5)
C2—C3	1.377 (3)	C12—H12	0.9300
C3—C4	1.378 (4)	C13—C14	1.384 (4)
C3—H3	0.9300	C13—H13	0.9300
C4—C5	1.373 (4)	C14—H14	0.9300
			
C7—N1—N2	114.9 (2)	N1—C7—H7	119.8
C8—N2—N1	119.05 (19)	C1—C7—H7	119.8
C8—N2—H2	123 (2)	O1—C8—N2	123.7 (2)
N1—N2—H2	118 (2)	O1—C8—C9	122.5 (2)
C2—C1—C6	116.7 (2)	N2—C8—C9	113.8 (2)
C2—C1—C7	121.8 (2)	C10—C9—C14	119.1 (2)
C6—C1—C7	121.5 (2)	C10—C9—C8	121.9 (2)
C3—C2—C1	122.3 (2)	C14—C9—C8	118.9 (2)
C3—C2—Cl1	117.5 (2)	C9—C10—C11	120.6 (3)
C1—C2—Cl1	120.12 (19)	C9—C10—Cl3	121.6 (2)
C2—C3—C4	118.6 (2)	C11—C10—Cl3	117.7 (2)
C2—C3—H3	120.7	C12—C11—C10	119.4 (3)
C4—C3—H3	120.7	C12—C11—H11	120.3
C5—C4—C3	121.1 (2)	C10—C11—H11	120.3
C5—C4—Cl2	120.4 (2)	C11—C12—C13	121.6 (3)
C3—C4—Cl2	118.4 (2)	C11—C12—H12	119.2
C6—C5—C4	119.3 (2)	C13—C12—H12	119.2
C6—C5—H5	120.4	C12—C13—C14	120.2 (3)
C4—C5—H5	120.4	C12—C13—H13	119.9
C5—C6—C1	122.0 (2)	C14—C13—H13	119.9
C5—C6—H6	119.0	C13—C14—C9	119.1 (3)
C1—C6—H6	119.0	C13—C14—H14	120.5
N1—C7—C1	120.4 (2)	C9—C14—H14	120.5

### Hydrogen-bond geometry (Å, °)

**Table:**

*D*—H···*A*	*D*—H	H···*A*	*D*···*A*	*D*—H···*A*
N2—H2···O1^i^	0.89 (1)	2.00 (1)	2.864 (3)	164 (3)

Symmetry codes: (i) *x*, −*y*+3/2, *z*−1/2.
